# Bioassay-Guided Phytochemical Investigation of Vietnamese *Vitex rotundifolia* Leaves and the Liverwort *Ptychanthus striatus* as Sources of SARS-CoV-2 Main Protease Inhibitors

**DOI:** 10.3390/molecules31122009

**Published:** 2026-06-08

**Authors:** Huy Truong Nguyen, Thi-Minh Dinh Tran, Thuc-Huy Duong, Trong-Hieu Bui, Nguyen-Kim-Tuyen Pham, Mai-Dang-Truong Pham, Hoang-Truc-Nguyen Phan, Dinh-Tri Mai, Warudee Pathummanee, Duc-Dung Pham, Tongsai Jamnongkan

**Affiliations:** 1 Research Group in Pharmaceutical and Biomedical Sciences, Faculty of Pharmacy, Ton Duc Thang University, Ho Chi Minh City 700000, Vietnam; nguyentruonghuy@tdtu.edu.vn; 2 Department of Biology, Ho Chi Minh City University of Education, 280 An Duong Vuong Street, Cho Quan Ward, Ho Chi Minh City 748342, Vietnam; dinhttm@hcmue.edu.vn; 3 Department of Chemistry, Ho Chi Minh City University of Education, 280 An Duong Vuong Street, Cho Quan Ward, Ho Chi Minh City 748342, Vietnam; huydt@hcmue.edu.vn (T.-H.D.); huybui2396@gmail.com (T.-H.B.); hn78786@gmail.com (H.-T.-N.P.); 4 Faculty of Engineering and Technology, Saigon University, Cho Quan Ward, Ho Chi Minh City 748342, Vietnam; phngktuyen@sgu.edu.vn; 5 Institute of Advanced Technology, Vietnam Academy of Science and Technology, Hanoi 100000, Vietnam; phammai.dangtruong@gmail.com; 6 Graduate University of Science and Technology, Vietnam Academy of Science and Technology, Hanoi 100000, Vietnam; maidinhtri@gmail.com; 7 Department of Fundamental Science and Physical Education, Faculty of Science at Sriracha, Kasetsart University, Chonburi 20230, Thailand; warudee.p@live.ku.th

**Keywords:** *Ptychanthus striatus*, *Vitex rotundifolia*, flavonoid, terpenoid, SARS-CoV-2 Mpro

## Abstract

*Vitex rotundifolia* is a medicinal plant rich in terpenoids and flavonoids, whereas the liverwort *Ptychanthus striatus* represents an underexplored bryophyte source of specialized metabolites. In this study, a bioassay-guided phytochemical investigation of Vietnamese *V. rotundifolia* leaves and *P. striatus* was conducted to identify natural inhibitors of SARS-CoV-2 main protease (Mpro). The crude methanol extracts and selected fractions showed inhibitory activity against SARS-CoV-2 Mpro, thereby guiding subsequent chromatographic separation. Thirteen compounds, including diterpenoids, lupane-type triterpenoids, and flavonoids, were isolated from *V. rotundifolia*, while ten terpenoid, phenolic, bibenzyl, and bisbibenzyl-type metabolites were obtained from *P. striatus*. Most isolated compounds are reported from these species for the first time, and compound **P8** from *P. striatus* is described as a new natural product. All isolated compounds were evaluated for their inhibitory activity against SARS-CoV-2 Mpro. Among them, chrysoplenol D was the most potent inhibitor, with an IC_50_ value of 0.08 ± 0.01 µM, followed by selected phenolic/bibenzyl-type metabolites from *P. striatus* and other flavonoid derivatives from *V. rotundifolia*. Most diterpenoids showed weak or negligible inhibition. Molecular docking studies supported the experimental results by showing that representative active compounds could bind within the catalytic pocket of SARS-CoV-2 Mpro and interact with key residues, including His41, Gly143, and Cys145. These findings expand the phytochemical knowledge of Vietnamese *V. rotundifolia* and *P. striatus* and highlight chrysoplenol D and related flavonoid or bibenzyl-type natural products as promising scaffolds for further development of SARS-CoV-2 Mpro inhibitors.

## 1. Introduction

Coronavirus disease 2019 (COVID-19), caused by severe acute respiratory syndrome coronavirus 2 (SARS-CoV-2), has remained a major global health concern and has continuously stimulated the search for effective antiviral agents [1,2,3]. Among the viral enzymes involved in this process, the SARS-CoV-2 main protease, also known as Mpro, 3CLpro, or nsp5, is considered a key antiviral target because it cleaves viral polyproteins at multiple conserved sites required for viral maturation and replication [4,5]. The discovery and clinical development of Mpro inhibitors have further validated this enzyme as a promising target for anti-SARS-CoV-2 therapy [6]. In particular, ensitrelvir has recently been approved as an orally active SARS-CoV-2 Mpro inhibitor, while other optimized Mpro inhibitors have shown improved antiviral profiles and reduced sensitivity to resistance-associated mutations [7,8]. Nevertheless, viral mutations, possible resistance, and the limitations of current antiviral treatments continue to justify the search for structurally diverse Mpro inhibitors with new chemical scaffolds [9,10]. Although clinically used or regionally approved SARS-CoV-2 Mpro inhibitors, such as nirmatrelvir/ritonavir, ensitrelvir, simnotrelvir/ritonavir, and leritrelvir, have validated Mpro as an important antiviral target, the discovery of additional scaffolds remains valuable. Continued viral evolution, possible resistance-associated mutations, drug–drug interaction concerns, and the need for backup chemotypes still justify further inhibitor discovery [6,10,11]. Therefore, natural products remain a valuable and complementary source of structurally diverse scaffolds for expanding the chemical space of SARS-CoV-2 Mpro inhibitors and supporting future antiviral lead optimization.

Natural products remain an important source of antiviral lead compounds because of their structural diversity and broad biological relevance. Various classes of natural metabolites, including alkaloids, flavonoids, phytosterols, terpenoids, phenolic compounds, and their derivatives, have been investigated as potential SARS-CoV-2 Mpro inhibitors using molecular docking, molecular dynamics, virtual screening, and biological assays [11,12,13,14]. For instance, kaempferol was evaluated as a potential inhibitor of SARS-CoV-2 Mpro through combined in silico and in vitro approaches, while secondary metabolites from *Mangifera indica* and antiviral medicinal herbs were reported to inhibit SARS-CoV-2 Mpro in target-based screening studies [11,13,14]. Other naturally occurring constituents, such as β-sitosterol and related terpenoid or sterol-type compounds, have also been proposed as potential Mpro binders in computational studies [15,16,17]. These studies indicate that medicinal plants and other underexplored natural sources may provide useful chemical scaffolds for the discovery of new Mpro inhibitors.

Recent investigations have also demonstrated the potential of Vietnamese biodiversity in the search for natural SARS-CoV-2 Mpro inhibitors. In particular, Le et al. (2025) reported natural inhibitors of SARS-CoV-2 Mpro from three Vietnamese bryophytes, namely *Erythrodontium julaceum*, *Marchantia polymorpha*, and *Plagiochila bantamensis* [18]. Notably, isoriccardin C and julacelide showed considerable inhibitory activity, with IC_50_ values of 15.7 and 2.84 µg/mL, respectively [18]. These findings suggest that Vietnamese bryophytes are underexplored but promising sources of structurally distinctive anti-Mpro metabolites. Therefore, the present study was designed as a continuation of our screening program aimed at identifying SARS-CoV-2 Mpro inhibitors from medicinal plants and bryophytes growing in Vietnam.

*Ptychanthus striatus* (Lehm. & Lindenb.) Nees, frequently encountered as a leafy liverwort within the Marchantiophyta division, is broadly distributed across the tropical and subtropical zones of Asia, Australasia, and Oceania. In Vietnam, it characteristically colonizes tree trunks, branches, and damp rock surfaces in humid, mountainous environments [18]. Historically, bryophytes—including liverworts and mosses—have occupied a notable place in ethnomedicine, where they are applied to treat a multitude of ailments [19]. Over the past few decades, phytochemical explorations of *Ptychanthus striatus* have revealed its thallus to be a prolific source of structurally intriguing secondary metabolites. Researchers have documented an extensive array of lipophilic compounds, primarily highlighting sesquiterpenoids, highly oxygenated labdane-type diterpenoids (such as the ptychantin series), and complex macrocyclic bis(bibenzyls) [20,21,22]. Among these, the unique terpenoid architectures and aromatic derivatives have drawn considerable interest from the scientific community as promising templates for natural product-based drug discovery [23]. Extracts and purified fractions of *Ptychanthus striatus* have been subjected to various biological evaluations, demonstrating a versatile pharmacological profile. Notably, recent findings indicate substantial antioxidant capabilities alongside potent inhibitory effects on *α*-amylase and *α*-glucosidase, pointing toward a meaningful antidiabetic potential [24]. Other investigations have shown that isolated bis(bibenzyls), such as ptychantol A, exhibit antitrypanosomal properties [25]. Additionally, the literature confirms that the chemical constituents of *Ptychanthus striatus* impart remarkable antimicrobial properties—particularly inhibiting *Candida* fungi and several bacterial strains—as well as pronounced cytotoxic effects that hinder the proliferation of specific tumor cell lines [26]. Despite its promising biological profiles, systematic studies elucidating the precise therapeutic mechanisms of *Ptychanthus striatus* are relatively limited compared to higher vascular plants. The existing literature predominantly focuses on the preliminary screening of crude extracts, thereby creating a critical gap in the functional validation of individual purified compounds. [27].

In parallel, *Vitex rotundifolia* L. was selected as a representative medicinal plant source. Medicinal plants have played a significant role in traditional healthcare systems for centuries, and the genus *Vitex* is among the most extensively studied groups within the family Verbenaceae. This genus comprises approximately 250 species distributed globally and has been utilized traditionally for the treatment of various ailments. Different parts of *Vitex* plants, including roots, stems, leaves, and fruits, have been employed in folk medicine, underscoring the extensive medicinal value of this genus [28]. *V. rotundifolia*, a coastal medicinal plant extensively distributed across Asian nations, stands as a prominent representative of this genus. Its use in traditional medicinal systems further supports its ethnopharmacological importance [29]. Previous phytochemical investigations of this species have unveiled a diverse array of secondary metabolites, encompassing diterpenoids, flavonoids, phenolics, phenolic glucosides, iridoid glucosides, norlignans, and polymethoxyflavonoids [30,31,32,33,34,35,36,37]. Recent studies have highlighted the pharmacological significance of the genus *Vitex*, particularly through investigations of its phytochemical constituents and potential therapeutic applications, including in the context of ischemic stroke [38]. In Vietnam, *V. rotundifolia* has garnered growing attention due to its potential medicinal properties. Recent research has focused on the chemical composition and biological activities of crude extracts derived from this plant [39]. Nevertheless, the chemical profiles of Vietnamese *V. rotundifolia* and *P. striatus* remain incompletely understood, particularly with regard to the constituents responsible for antiviral or SARS-CoV-2 Mpro inhibitory activity. Given their distinct phytochemical characteristics, further bioassay-guided investigation of these two *Vietnamese* natural sources is required to identify active metabolites and expand the chemical diversity of natural Mpro inhibitors.

**Figure 1:**
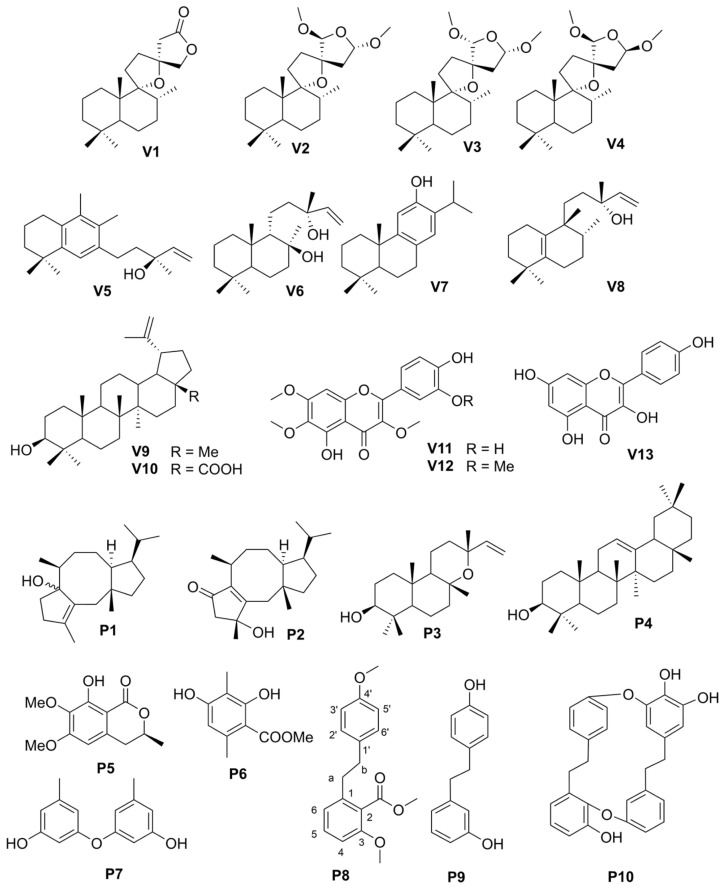
Chemical structures of **V1**–**V13** and **P1**–**P10**.

Based on the aforementioned considerations, the present study investigated *V. rotundifolia* and *P. striatus* as two chemically distinct Vietnamese natural sources for the discovery of SARS-CoV-2 Mpro inhibitors. *V. rotundifolia* stands as a medicinal vascular plant characterized by its abundance of terpenoids and flavonoid-related compounds. On the other hand, *P. striatus* emerges as an underexplored liverwort source of specialized bryophyte metabolites, particularly terpenoids and bisbibenzyl-type compounds. Through a bioassay-guided phytochemical investigation, this study aimed to isolate and characterize secondary metabolites from the two species and evaluate their inhibitory activity against SARS-CoV-2 Mpro (Figure 1). The findings are expected to expand the chemical diversity of natural Mpro inhibitors and further highlight the potential of Vietnamese medicinal plants and bryophytes as promising sources for antiviral natural-product discovery.

## 2. Results

### 2.1. SARS-CoV-2 Mpro Inhibition of Extracts and Fractions of Vitex rotundifolia and Ptychanthus striatus

**Figure 2:**
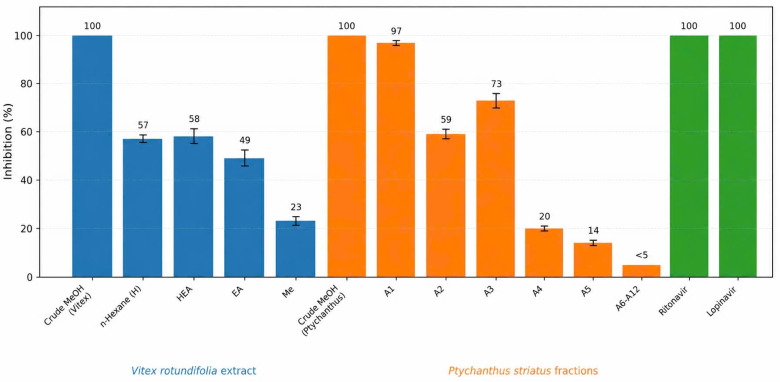
Inhibitory activity (%) of extracts and fractions from *Vitex rotundifolia* and *Ptychanthus striatus* at 100 µg/mL against SARS-CoV-2 Mpro. All experiments were performed in triplicate, and the results are expressed as mean ± standard deviation (SD).

The crude MeOH extracts of *Vitex rotundifolia* and *Ptychanthus striatus* showed strong inhibitory activity against SARS-CoV-2 Mpro, indicating that both materials contained bioactive constituents (Figure 2). After solvent partition and chromatographic fractionation, the activity was not evenly distributed among the fractions. For *V. rotundifolia*, the *n*-hexane and HEA fractions retained higher activity than the more polar fractions, suggesting that the active constituents were mainly concentrated in the less polar to medium-polar fractions (Figure 2). For *P. striatus*, fraction A1 showed the strongest inhibition, followed by A3 and A2, whereas the remaining fractions were weakly active or inactive. Based on this activity profile, the most active fractions were selected for further phytochemical investigation. This bioassay-guided approach led to the isolation of structurally diverse metabolites, including diterpenoids, triterpenoids, flavonoids, phenolic derivatives, and bibenzyl-type compounds, which were subsequently evaluated to identify the compounds responsible for the observed Mpro inhibitory activity.

### 2.2. Phytochemical Investigation of Vitex rotundifolia and Ptychanthus striatus

Using bio-guided procedure, the leaves of the medicinal plant *Vitex rotundifolia* and the leafy liverwort *Ptychanthus striatus* were subjected to phytochemical investigation. Phytochemical investigation of the most active extracts (H and HEA extracts) of *Vitex rotundifolia* led to the isolation and identification of thirteen secondary metabolites, designated as **V1**–**V13**. These compounds were identified as previtexilactone (**V1**) [40], (13*S*,15*S*,16*R*)-9,13,15,16-diepoxy-15,16-dimethoxylabdane (**V2**) [35], (13*S*,15*R*,16*S*)-9,13,15,16-diepoxy-15,16-dimethoxylabdane (**V3**) [35], (13*R*,15*R*,16*S*)-9,13,15,16-diepoxy-15,16-dimethoxylabdane (**V4**) [35], (+)-isofregenedol (**V5**) [41], sclareol (**V6**) [40], ferruginol (**V7**) [42], *cis*-3,14-clerodadien-13-ol (**V8**) [43], lupeol (**V9**)**,** betulinic acid (**V10**) [44], chrysoplenol D (**V11**) [45], artemetin (**V12**) [45], and kaempferol (**V13**) [45]. In parallel, repeated chromatographic separation of the most active fractions **A1**–**A3** of *Ptychanthus striatus* afforded ten secondary metabolites, designated as **P1**–**P10**. These compounds were identified as fusicoauritone (**P1**) [46], (1*R*,4*S*,6a*S*,7*R*,9a*R*)-1-hydroxy-7-isopropyl-1,4,9a-trimethyl-1,4,5,6,6a,7,8,9,9a,10-decahydrodicyclopenta[a,d][8]annulen-3(2H)-one (**P2**) [47], *β*-amyrin (**P3**) [48]**,** 3*β*-hydroxymanoyloxide (**P4**) [49], (+)-kigelin (**P5**) [50,51], methyl *β*-orsellinate (**P6**) [52], diorcinol (**P7**) [53], methyl 2-methoxy-6-[2-(4-methoxyphenyl)ethyl]benzoate (**P8**) [54], lununarin (**P9**) [55], and marchantin A (**P10**) [55]. The structures of the known compounds were elucidated by spectroscopic analysis and comparison with literature data reported for the corresponding identical compounds. NMR data of compounds **P5** and **P8** were described as follows:

### 2.3. SARS-CoV-2 Mpro Inhibitory Activity of Isolated Compounds

**Figure 3:**
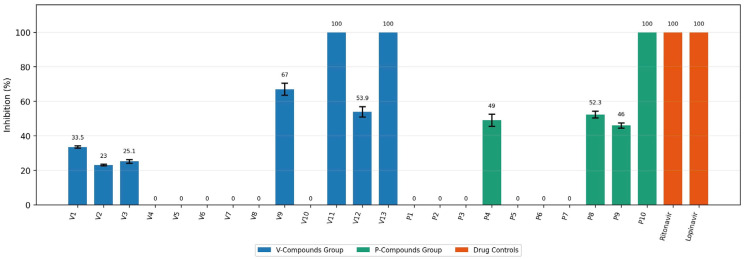
Inhibitory activity of compounds **V1**–**V13** and **P1**–**P10** against SARS-CoV-2 Mpro at 100 µg/mL. All experiments were performed in triplicate, and the results are expressed as mean ± standard deviation (SD).

**Figure 4:**
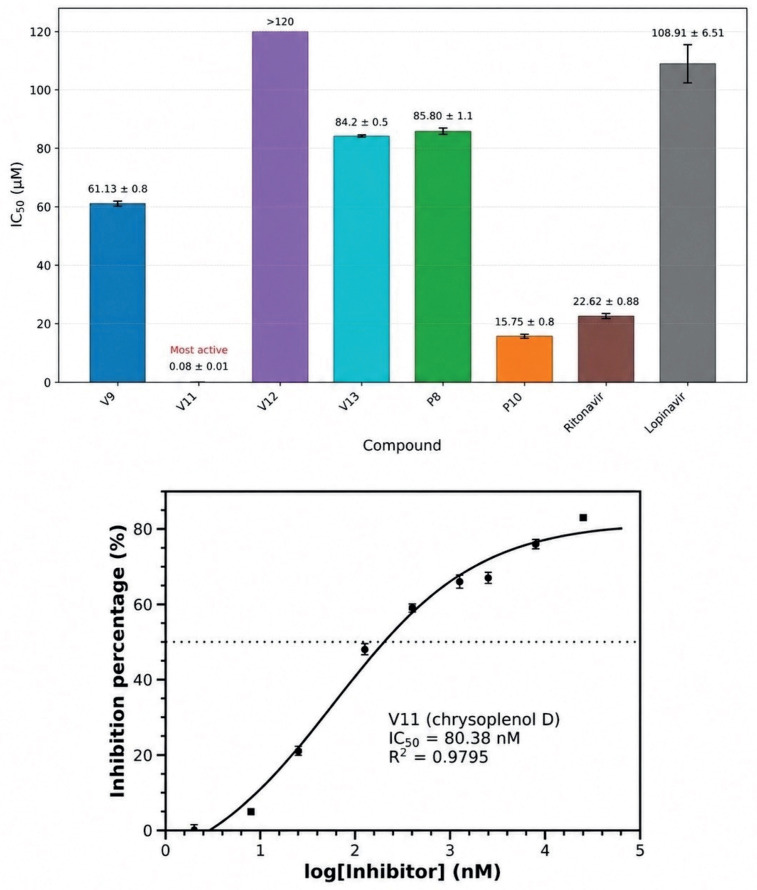
IC_50_ values of selected compounds against SARS-CoV-2 Mpro.

The isolated compounds were first screened for SARS-CoV-2 Mpro inhibitory activity at 100 µg/mL to compare their relative potency and to identify the most active constituents (Figure 3). The results showed clear differences in activity according to both carbon skeleton and substitution pattern. Based on the screening results, the most active compounds were selected for IC_50_ determination (Figure 4). Chrysoplenol D (**V11**) was the most potent inhibitor, with an IC_50_ value of 0.08 ± 0.01 µM, followed by **P8**, a bibenzyl-type derivative from *P. striatus* with an IC_50_ value of 15.75 ± 0.8 µM. Lupeol (**V9**) and kaempferol (**V13**) showed weaker but measurable inhibition, with IC_50_ values of 61.13 ± 0.8 and 84.2 ± 0.5 µM, respectively. The outstanding inhibitory potency of chrysoplenol D (**V11**) may be attributed to its ability to establish a greater number of hydrogen-bond interactions with key residues in the Mpro active site compared with the other investigated compounds. This extensive hydrogen-bonding network enhanced the stability of the protein–ligand complex, thereby contributing to its superior in vitro inhibitory activity.

### 2.4. Molecular Docking Studies

The redocking protocol was successfully validated for the holo-state Mpro structure (PDB ID: 9HAK), resolved at 1.25 Å, which is complexed with its native ligand (A1ITI). The redocking protocol was successfully validated for the Mpro structure (PDB ID: 9HAK), with an RMSD value of 0.8 Å for the protein–ligand complex relative to the native ligand, satisfying the acceptance criterion (RMSD < 2 Å). Next, molecular docking was carried out for four ligands (**P8**, **V9**, **V11**, and **V13**) against the Mpro structure (PDB ID: 9HAK) using Glide (SP) (Table 1). The obtained docking scores spanned from −4.4 to −7.8 kcal/mol, while the corresponding MM-GBSA binding free energies ranged from −50.6 to −65.0 kcal/mol. For context, the native ligand showed values of −9.3 kcal/mol (docking score) and −108.9 kcal/mol (MM-GBSA). Thus, the calculated binding energies of the studied ligands correspond to approximately 45–80% of those of the native ligand, depending on the evaluation method. Importantly, the binding modes of these ligands preserved several key interactions within the active site. In particular, contacts with Gly143 (**V11**, **P8**), Cys145 (**V11**, **P8**), and His41 (**V13**, **P8**) were consistently observed, suggesting that the compounds adopt orientations compatible with the catalytic environment.

## 3. Discussion

### 3.1. Phytochemical Diversity and Structural Elucidation

The isolates from *V. rotundifolia* represent several major structural classes. Compounds **V1**–**V6** are diterpenoids, including labdane-type and related oxygenated diterpene derivatives, while **V7** is an abietane-type diterpenoid and **V8** is a clerodane-type diterpenoid. Compounds **V9** and **V10** are lupane-type triterpenoids, whereas **V11**–**V13** are flavonoid derivatives. This chemical profile is consistent with previous phytochemical studies of the genus *Vitex*, which is known to produce structurally diverse terpenoids and flavonoids. Notably, compounds **V1**, **V4**–**V8**, and **V10** are reported here for the first time from *V. rotundifolia*, thereby expanding the known chemical diversity of this medicinal species. The occurrence of multiple diterpenoid skeletons, together with lupane triterpenoids and polymethoxylated flavonoids, further supports *V. rotundifolia* as a chemically rich source of bioactive natural products. The metabolites isolated from *P. striatus* display considerable structural diversity, including sesquiterpenoid- and diterpenoid-related terpenes, triterpenoids, phenolic esters, bibenzyl derivatives, and macrocyclic bisbibenzyl-type constituents. Such diversity is characteristic of liverworts, which are well known for producing lipophilic terpenoids and aromatic metabolites. Among the isolated compounds, **P1**, **P3**–**P10** are reported here for the first time from *P. striatus*. Moreover, these compounds also appear to represent their first report from the genus *Ptychanthus*. Compound **P8**, methyl 2-methoxy-6-[2-(4-methoxyphenyl)ethyl]benzoate, has not been previously reported as a natural product, and is therefore proposed as a new natural compound. These findings expand the phytochemical knowledge of Vietnamese *P. striatus* and provide a basis for further biological evaluation of this liverwort. Overall, the present phytochemical study revealed that *V. rotundifolia* and *P. striatus* are two chemically distinct natural sources. *V. rotundifolia* mainly yielded diterpenoids, triterpenoids, and flavonoids, whereas *P. striatus* afforded a broader set of liverwort-associated terpenoid and aromatic metabolites. The isolation of several compounds newly reported from these species, together with a putative new natural compound from *P. striatus*, highlights the value of Vietnamese medicinal plants and liverworts as sources of structurally diverse secondary metabolites. These isolated compounds provide an important foundation for evaluating their inhibitory activity against SARS-CoV-2 Mpro and for identifying the chemical constituents responsible for the bioactivity observed in the crude extracts.

**Figure 5:**
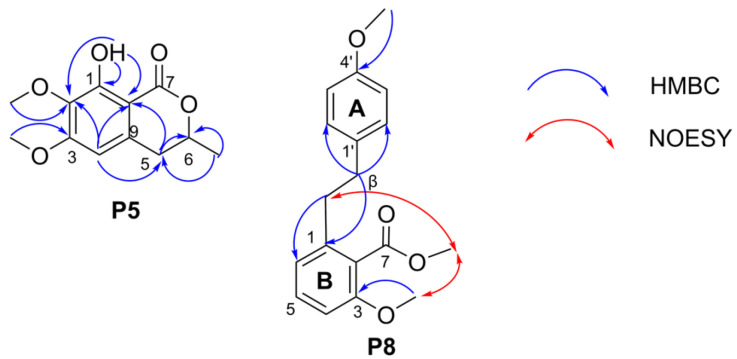
Chemical structures and selected HMBC and NOESY correlations of compounds **P5** and **P8**.

The NMR data of **P5** were in good agreement with those reported for kigelin (Figure 5 and Table 2). Accordingly, compound **P5** was identified as kigelin [56]. This compound was first isolated from the roots and stem bark of *Kigelia pinnata* (Bignoniaceae) by Govindachari and co-workers in 1971. The authors defined the 6*R* configuration based on the negative optical rotation. Markad and co-workers synthesized (−)-kigelin using multiple transformed steps to confirm its absolute configuration. In our scenario, the positive specific rotation of **P5** is +137 (*c* 0.1, MeOH), indicating that it might have a 6*S* configuration, in contrast to previously reported (−)-kigelin, and supporting its assignment as an enantiomer of (−)-kigelin.

The ^1^H NMR spectrum of compound **P8** displayed signals characteristic of two aromatic rings. The first aromatic ring (ring A) was assigned as a 1′,4′-disubstituted benzene unit, as indicated by two pairs of ortho-coupled aromatic protons at *δ*_H_ 7.10 (2H, *d*, *J* = 8.5 Hz, H-2′/H-6′) and 6.84 (2H, *d*, *J* = 8.5 Hz, H-3′/H-5′). The second aromatic ring (ring B) showed a 1,2,3-trisubstituted substitution pattern, supported by three aromatic proton signals at *δ*_H_ 6.79 (1H, *d*, *J* = 8.0 Hz, H-4), 6.80 (1H, *d*, *J* = 8.0 Hz, H-6), and 7.28 (1H, *d*, *J* = 8.5 Hz, H-5). In addition, two methylene groups were observed at *δ*_H_ 2.82–2.83 (4H, m, H-*α*/H-*β*), together with three methoxy singlets at *δ*_H_ 3.94 (3H, s, 7-OCH_3_), 3.85 (3H, s, 3-OCH_3_), and 3.80 (3H, s, 4′-OCH_3_). The ^13^C-NMR (JMOD) and HMQC spectra further supported the proposed structure by showing five aromatic methine carbons at *δ*_C_ 108.9 (C-4), 130.5 (C-5), 121.8 (C-6), 129.4 (C-2′/C-6′), and 113.9 (C-3′/C-5′), two methylene carbons at *δ*_C_ 36.2 (C-α) and 36.9 (C-*β*), and three oxygenated methyl carbons at *δ*_C_ 56.1 (3-OCH_3_), 55.4 (4′-OCH_3_), and 52.4 (7-OCH_3_). The presence of a carbonyl carbon at *δ*_C_ 169.7, together with the methoxy resonance at *δ*_H_ 3.94/*δ*_C_ 52.4, indicated the presence of a methyl ester group. The substitution pattern and connectivities of **P8** were established by analysis of the HMBC correlations. The 1′,4′-disubstituted aromatic ring A was confirmed by HMBC correlations from H-2′/H-6′ (*δ*_H_ 7.10) and H-3′/H-5′ (*δ*_H_ 6.84) to C-4′ (*δ*_C_ 157.9). The methoxy group at *δ*_H_ 3.80 showed a clear HMBC correlation with C-4′, confirming the location of the 4′-OCH_3_ group (Figure 5). The correlation from H-2′/H-6′ to C-*β* (*δ*_C_ 36.9) established the connection between ring A and the β-methylene group. For ring B, HMBC correlations from H-6 (*δ*_H_ 6.80) to C-α (*δ*_C_ 36.2) and C-4 (*δ*_C_ 108.9), together with correlations from H-5 (*δ*_H_ 7.28) to C-1 (*δ*_C_ 140.2) and C-3 (*δ*_C_ 156.8), supported the 1,2,3-trisubstituted aromatic system and the attachment of ring B to the α-methylene group. The methoxy proton signal at *δ*_H_ 3.94 correlated with the carbonyl carbon C-7 (*δ*_C_ 169.7), confirming the methyl ester functionality, while the 3-OCH_3_ signal at δ_H_ 3.85 showed an HMBC correlation with C-3 (*δ*_C_ 156.8), establishing the methoxy substitution at C-3. Furthermore, correlations from H-*α* to C-6, from H-*β* to C-2′/C-6′, and from H-*α*/H-*β* to C-1′ and C-1 confirmed that the two aromatic rings were connected through a dimethylene bridge. The NOESY spectrum provided additional support for the substitution pattern and spatial arrangement of **P8**. The NOESY correlation between H-*α* (*δ*_H_ 2.83) and H-6 (*δ*_H_ 6.80), together with the correlation involving 7-OCH_3_ (*δ*_H_ 3.94), indicated spatial proximity between the *α*-methylene group and the substituted aromatic ring B. The NOESY correlation between H-*β* (*δ*_H_ 2.83) and H-6′ (*δ*_H_ 7.10) supported the attachment of the β-methylene group to ring A. In addition, the correlations of 4′-OCH_3_ (δ_H_ 3.80) with H-5′ (*δ*_H_ 6.84) and of 3-OCH_3_ (*δ*_H_ 3.85) with H-4 (*δ*_H_ 6.79) further confirmed the positions of the methoxy groups on rings A and B, respectively. Comparison of the NMR data of **P8** with those of lunularic acid revealed close structural similarity (Table 3). The main difference was the presence of three methoxy groups in **P8**, corresponding to methyl esterification and two aromatic methoxy substituents. Accordingly, compound **P8** was identified as methyl 2-methoxy-6-[2-(4-methoxyphenyl)ethyl]benzoate. This compound was previously synthesized by Azzena and co-workers in 1995; however, its NMR spectroscopic data were not reported. The molecular formula of **P8** was confirmed as C_18_H_20_O_4_ based on its ESI(+) mass spectrum, which showed sodiated and protonated ion peaks at m/z 323.1 [M+Na]^+^ and 301.1 [M+H]^+^, respectively, consistent with the calculated values for C_18_H_20_O_4_Na^+^ (calcd. 323.1) and C_18_H_21_O_4_^+^ (calcd. 301.1) (Supplementary Materials Figure S6). To the best of our knowledge, **P8** has not previously been isolated from a natural source. Therefore, compound **P8** is described here as a new natural compound.

### 3.2. Biological Activity

The isolated compounds were first screened for SARS-CoV-2 Mpro inhibitory activity at 100 µg/mL to compare their relative potency and to identify the most active constituents. The results showed clear differences in activity according to both carbon skeleton and substitution pattern. Among the compounds isolated from *Vitex rotundifolia*, most diterpenoids showed weak or negligible inhibition. Previtexilactone (**V1**) and 9,13,15,16-diepoxy-15,16-dimethoxylabdanes (**V2** and **V3**) displayed only low to moderate activity, whereas the related labdane-type derivative (13*R*,15*R*,16*S*)-9,13,15,16-diepoxy-15,16-dimethoxylabdane (**V4**), (+)-isofregenedol (**V5**), and sclareol (**V6**) were inactive or nearly inactive. Similarly, the abietane-type diterpenoid ferruginol (**V7**) and the clerodane-type diterpenoid *cis*-3,14-clerodadien-13-ol (**V8**) did not exhibit significant inhibition. This trend suggests that the diterpenoid-type skeletons, including labdane, abietane, and clerodane skeletons, were generally not favorable for Mpro inhibition. In contrast, the lupane-type triterpenoid lupeol (**V9**) showed stronger activity than the diterpenoids, indicating that a larger pentacyclic triterpenoid carbon framework may provide a more favorable scaffold. However, betulinic acid (**V10**), another lupane-type triterpenoid, was less active, suggesting that changes in the substituent pattern on the lupane skeleton can markedly affect activity.

The strongest activity among the *V. rotundifolia* isolates was observed for the flavonoid derivatives. Chrysoplenol D (**V11**) and kaempferol (**V13**) showed the highest inhibition in the initial screening, whereas artemetin (**V12**) was much weaker. This comparison indicates that the flavonoid carbon framework is more favorable for Mpro inhibition than the diterpenoid skeletons in this series.

For the compounds isolated from *Ptychanthus striatus*, most terpenoid and simple aromatic metabolites showed weak or no inhibition in the 100 µg/mL screening. Fusicoauritone (**P1**), (1*R*,4*S*,6a*S*,7*R*,9a*R*)-1-hydroxy-7-isopropyl-1,4,9a-trimethyl-1,4,5,6,6a,7,8,9,9a,10-decahydrodicyclopenta[a,d][8]annulen-3(2*H*)-one (**P2**), β-amyrin (**P3**), 3*β*-hydroxymanoyloxide (**P4**), (+)-kigelin (**P5**), methyl 2-methoxy-6-[2-(4-methoxyphenyl)ethyl]benzoate (**P8**), and methyl *β*-orsellinate (**P6**) were inactive or only weakly active, although 3*β*-hydroxymanoyloxide (**P4**) showed moderate inhibition. Diorcinol (**P7**), lunularin (**P9**), and marchantin A (**P10)** showed weak to moderate activity.

Based on the screening results, the most active compounds were selected for IC_50_ determination. Chrysoplenol D (**V11**) was the most potent inhibitor, with an IC_50_ value of 0.08 ± 0.01 µM, followed by the active phenolic/bibenzyl-type compound from *P. striatus* with an IC_50_ value of 15.75 ± 0.8 µM. Lupeol (**V9**) and kaempferol (**V13**) showed weaker but measurable inhibition, with IC_50_ values of 61.13 ± 0.8 and 84.2 ± 0.5 µM, respectively. Overall, the activity profile suggests that SARS-CoV-2 Mpro inhibition in this study is mainly associated with selected flavonoid and phenolic/bibenzyl-type frameworks, whereas the diterpenoid skeletons were generally less favorable. These results further indicate that both the carbon framework and substituent pattern play important roles in the observed activity. This observation is also consistent with previous reports showing that flavonoids can inhibit SARS-CoV-2 Mpro and that their activity is strongly influenced by hydroxylation, methoxylation, and other substituent patterns [13,57,58]. Khan et al. (2021) reported that kaempferol can interact with key residues in the Mpro active site and inhibit the enzyme in vitro [13], supporting our finding that kaempferol (**V13**) showed measurable but moderate inhibition, with an IC_50_ value of 84.2 ± 0.5 µM. Compared with chrysoplenol D, the weaker activity of kaempferol may be related to differences in substitution pattern. While kaempferol contains multiple free hydroxy groups, chrysoplenol D possesses both hydroxy and methoxy substituents, which may provide a more favorable balance between hydrogen-bonding capacity and hydrophobic interactions within the Mpro catalytic pocket [13]. The importance of flavonoid structural variation was further supported by Lin et al. (2023) [57], who screened 1019 plant flavonoids against SARS-CoV-2 Mpro and showed that activity depends strongly on flavonoid subclass and substitution pattern, with galloylated flavonoids, biflavones, and selected aglycones being enriched among active compounds. Toigo et al. (2023) also noted that quercetin, kaempferol, luteolin, baicalein, and related flavonoids are frequently investigated against COVID-19-related targets, including Mpro/3CLpro, PLpro, RdRp, Spike protein, and ACE2 [58]. This agrees with our results, in which chrysoplenol D was much more potent than kaempferol and artemetin despite their related flavonoid frameworks. The difference among chrysoplenol D, artemetin, and kaempferol also shows that the substitution pattern on the flavonoid nucleus is important. Chrysoplenol D contains both hydroxy and methoxy substituents, kaempferol contains multiple hydroxy groups, whereas artemetin is more highly methoxylated. The lower activity of artemetin suggests that extensive methoxylation on the flavonoid skeleton may reduce inhibitory activity, while a more suitable balance between hydroxy and methoxy groups appears to be beneficial. In the present study, the docking results further supported the experimental activity of chrysoplenol D, showing a favorable docking score and multiple hydrogen-bonding interactions with residues located in or near the catalytic region, including Gly143 and Cys145. However, because much of the evidence remains computational or limited to biochemical assays, the strong IC_50_ value of chrysoplenol D should still be interpreted cautiously until its cytotoxicity, selectivity, pharmacokinetic properties, and cell-based antiviral activity are confirmed.

**Figure 6:**
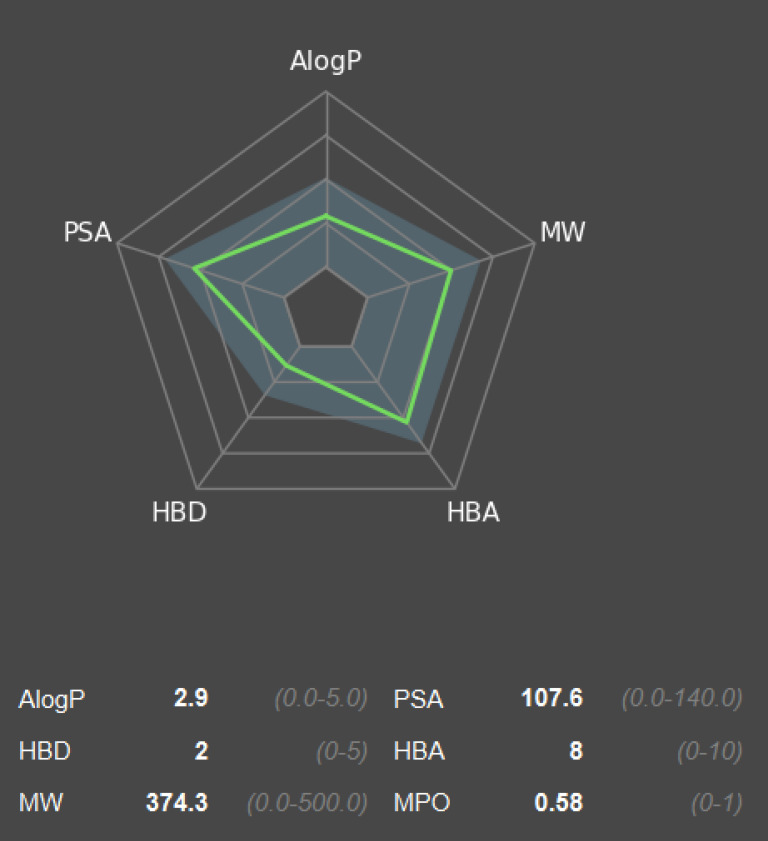
Drug-Likeness and Physicochemical Properties (Lipinski’s Rule of 5) of **V11**.

SARS-CoV-2 Mpro is a validated antiviral target because it plays an essential role in viral polyprotein processing and has no close human homologues with the same cleavage specificity, making it attractive for inhibitor development [4,5]. Nevertheless, potent inhibition in a biochemical FRET-based assay does not necessarily indicate cellular antiviral efficacy or drug-like potential. Although chrysoplenol D (**V11**) showed the strongest SARS-CoV-2 Mpro inhibitory activity in the present enzyme-based assay, its biological relevance should be interpreted with caution. At this stage, chrysoplenol D should be regarded as a promising in vitro Mpro inhibitory hit rather than a confirmed antiviral lead. Additional studies are required to determine its physicochemical properties, aqueous solubility, membrane permeability, metabolic stability, and cytotoxicity in relevant non-cancerous and virus-permissive cell lines. The predicted physicochemical properties of chrysoplenol D, calculated using Maestro (Schrödinger), strictly comply with Lipinski’s Rule of Five (Ro5), which is widely used to evaluate oral bioavailability [59]. As shown in the radar plot (Figure 6), all parameters fall within the optimal drug-like chemical space. Chrysoplenol D has a molecular weight of 374.3 Da, which is below the recommended threshold of 500 Da. This relatively low molecular weight may favor permeability and aqueous solubility [60]. Its calculated logP value is 2.9, which falls within the acceptable range of 0.0–5.0. This intermediate lipophilicity suggests a favorable balance between membrane permeability and aqueous solubility, while reducing the risk of non-specific binding or metabolic instability that is often associated with highly hydrophobic compounds [61]. Chrysoplenol D also contains two hydrogen-bond donors and eight hydrogen-bond acceptors, meeting the Ro5 criteria of ≤5 hydrogen-bond donors and ≤10 hydrogen-bond acceptors. Its polar surface area (PSA) is 107.6 Å^2^, which is below the commonly accepted threshold of 140 Å^2^, and is therefore compatible with favorable intestinal epithelial permeability and oral absorption [60,61]. Furthermore, the multiparameter optimization (MPO) score of chrysoplenol D is 0.58 on a scale of 0 to 1, suggesting a balanced physicochemical profile suitable for further lead optimization [62]. However, these predicted properties should not be overinterpreted. Although the physicochemical profile of chrysoplenol D appears favorable, structural features such as phenolic hydroxy and methoxy groups may also influence solubility, membrane permeability, and metabolic stability. In particular, phenolic hydroxy groups may undergo phase-II conjugation, whereas methoxy groups may be susceptible to oxidative metabolism. Therefore, the metabolic stability and pharmacokinetic behavior of chrysoplenol D require further experimental evaluation. The present study did not evaluate cytotoxicity in mammalian host cells, selectivity over human proteases, or activity against other SARS-CoV-2 enzymes such as PLpro or RdRp. Because enzyme inhibition does not necessarily translate into cellular antiviral efficacy, future studies should include cytotoxicity assays in relevant host-cell lines, cell-based SARS-CoV-2 replication assays, determination of EC_50_ and CC_50_ values, and calculation of a selectivity index. Additional counter-screening against host proteases and other viral enzymes would also be necessary to clarify whether chrysoplenol D acts selectively on SARS-CoV-2 Mpro. Thus, while the low IC_50_ value and docking interactions support chrysoplenol D as an interesting Mpro-targeting scaffold, further ADME, selectivity, cytotoxicity, and cellular antiviral studies are required before it can be advanced as a realistic antiviral lead.

## 4. Materials and Methods

### 4.1. Plant and Liverwort Materials

The leaves of *Vitex rotundifolia* were collected in Binh Thuan Province, Vietnam, in April 2025. The plant material was authenticated by Assoc. Prof. Dr. Dang Van Son, Institute of Tropical Biology, Vietnam. A voucher specimen was deposited under the code UE-P021 at the Natural Products Research Laboratory, Department of Organic Chemistry, Faculty of Chemistry, Ho Chi Minh City University of Education, Vietnam. The liverwort *Ptychanthus striatus* was collected in Lam Dong Province, Vietnam, in April 2025, and was identified by Dr. Tram Nguyen Khanh Trinh, Faculty of Biology, University of Science, Vietnam National University Ho Chi Minh City. A voucher specimen was deposited under the code UE-LW02 at the Natural Products Research Laboratory, Department of Organic Chemistry, Faculty of Chemistry, Ho Chi Minh City University of Education, Vietnam.

All chemicals including *n*-hexane, ethyl acetate, ethanol, dichloromethane, methanol were obtained by ChemSol Co. Ltd. (Hochiminh city, Viet Nam).

### 4.2. Isolation Procedure

**Figure 7:**
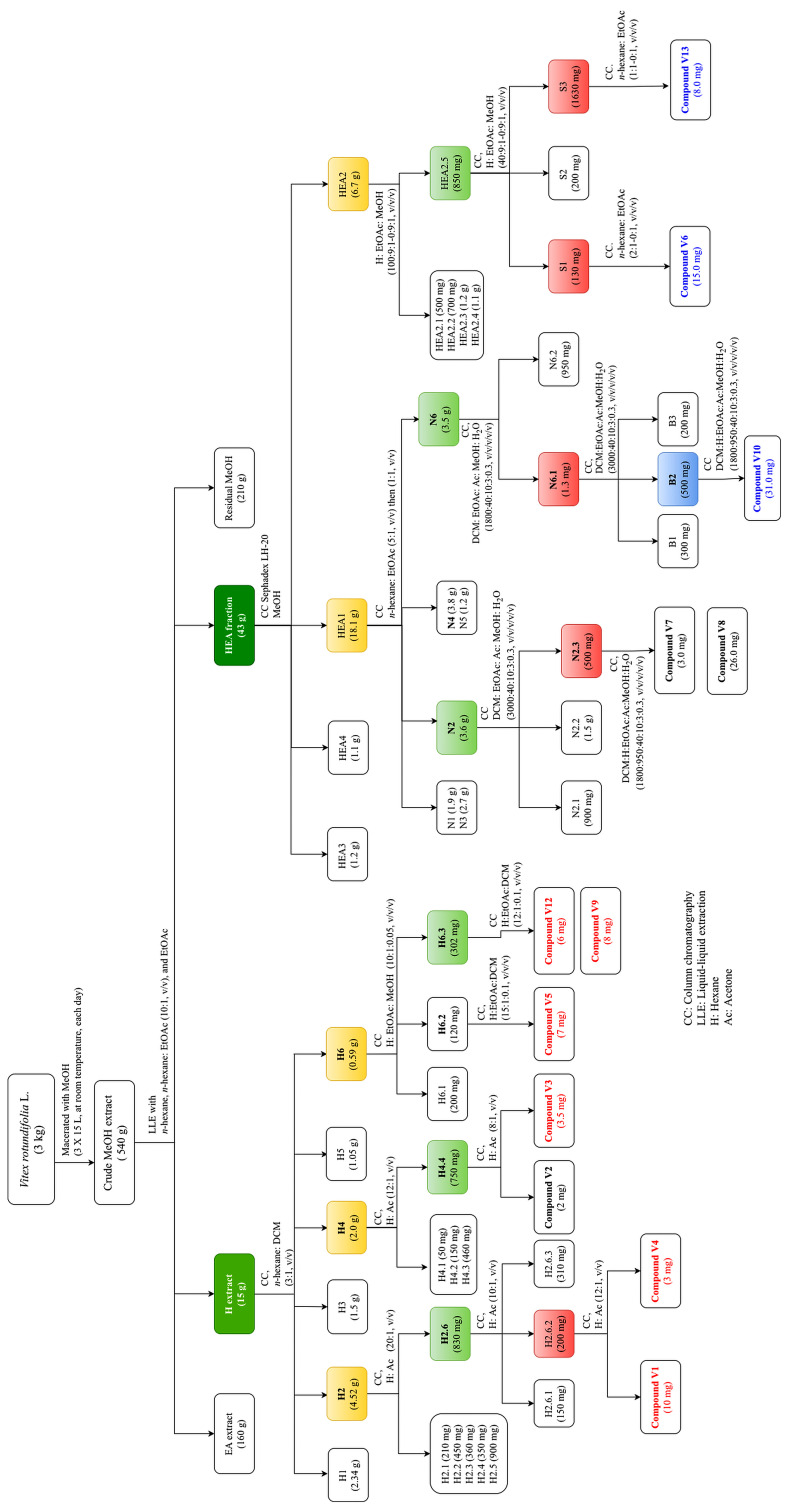
Isolation procedure of compounds **V1**–**V13**.

The dried leaves of *Vitex rotundifolia* L. (3.0 kg) were macerated with MeOH at room temperature (3 × 15 L, each day) to afford a crude MeOH extract (540 g) after removal of the solvent under reduced pressure. The crude MeOH extract was suspended in water and successively partitioned by liquid–liquid extraction with *n*-hexane, *n*-hexane–EtOAc (10:1, *v*/*v*), and EtOAc to yield the *n*-hexane extract (H, 15 g), *n*-hexane–EtOAc extract (HEA, 43 g), EtOAc extract (EA, 160 g), and water-soluble MeOH residue (210 g). The H fraction (15 g) was subjected to silica gel column chromatography using *n*-hexane–CH_2_Cl_2_ (3:1, *v*/*v*) to give six fractions, H1–H6. Fraction H2 (4.52 g) was chromatographed over silica gel with *n*-hexane–acetone (20:1, *v*/*v*) to afford fractions H2.1–H2.6. Fraction H2.6 (830 mg) was further separated using *n*-hexane–acetone (10:1, *v*/*v*) to give fractions H2.6.1–H2.6.3. Fraction H2.6.2 (200 mg) was purified by silica gel column chromatography with *n*-hexane–acetone (12:1, *v*/*v*) to afford compounds **V1** (10 mg) and **V4** (3 mg). Fraction H4 (2.0 g) was separated by silica gel column chromatography using *n*-hexane–acetone (12:1, *v*/*v*) to yield fractions H4.1–H4.4. Fraction H4.4 (750 mg) was further purified with *n*-hexane–acetone (8:1, *v*/*v*) to afford compounds **V2** (2 mg) and **V3** (3.5 mg). Fraction H6 (0.59 g) was chromatographed using *n*-hexane–EtOAc–MeOH (10:1:0.05, *v*/*v*/*v*) to give fractions H6.1–H6.3. Fraction H6.2 (120 mg) was purified by column chromatography using *n*-hexane–EtOAc–CH_2_Cl_2_ (15:1:0.1, *v*/*v*/*v*) to obtain compound **V5** (7 mg). Fraction H6.3 (502 mg) was further separated with *n*-hexane–EtOAc–CH_2_Cl_2_ (12:1:0.1, *v*/*v*/*v*) to yield compounds **V12** (6 mg) and **V9** (8 mg). The HEA fraction (43 g) was fractionated over Sephadex LH-20 using MeOH to give four fractions, HEA1–HEA4. Fraction HEA1 (18.1 g) was subjected to silica gel column chromatography with *n*-hexane–EtOAc (5:1 to 1:1, *v*/*v*) to afford six fractions, N1–N6. Fraction N2 (3.6 g) was chromatographed using CH_2_Cl_2_–EtOAc–acetone–MeOH–H_2_O (3000:40:10:3:0.3, *v*/*v*/*v*/*v*/*v*) to yield fractions N2.1 and N2.2. Fraction N3 (2.7 g) gave fraction N3.3 (500 mg), which was further purified by column chromatography with CH_2_Cl_2_–*n*-hexane–EtOAc–acetone–MeOH–H_2_O (1800:950:40:10:3:0.3, *v*/*v*/*v*/*v*/*v*/*v*) to afford compounds **V7** (3.0 mg) and **V8** (26.0 mg). Fraction N4 (3.8 g) afforded compound **V11** (15.0 mg). Fraction N6 (3.5 g) was purified using CH_2_Cl_2_–EtOAc–acetone–MeOH–H_2_O (1800:40:10:3:0.3, *v*/*v*/*v*/*v*/*v*) to give fractions N6.1 and N6.2. Fraction N6.1 (1.3 mg) was further separated with CH_2_Cl_2_–EtOAc–acetone–MeOH–H_2_O (3000:40:10:3:0.3, *v*/*v*/*v*/*v*/*v*) to yield fractions B1–B3. Fraction B2 (500 mg) was subsequently chromatographed with CH_2_Cl_2_–*n*-hexane–EtOAc–acetone–MeOH–H_2_O (1800:950:40:10:3:0.3, *v*/*v*/*v*/*v*/*v*/*v*) to afford compound **V10** (31.0 mg). Fraction HEA2 (6.7 g) was separated by silica gel column chromatography using *n*-hexane–EtOAc–MeOH (100:9:1-0:9:1, *v*/*v*/*v*) to yield fractions HEA2.1–HEA2.5. Fraction HEA2.5 (850 mg) was further purified by column chromatography with *n*-hexane–EtOAc–MeOH (40:9:1:–0:9:1, *v*/*v*/*v*) to give fractions S1–S3. Fraction S1 (130 mg) was purified by column chromatography with *n*-hexane–EtOAc (2:1:0.1, *v*/*v*/*v*) to yield compound **V6** (15.0 mg), whereas fraction S3 (630 mg) was purified with *n*-hexane–EtOAc (1:1:0.1, *v*/*v*/*v*) to afford compound **V13** (8.0 mg) (Figure 7).

**Figure 8:**
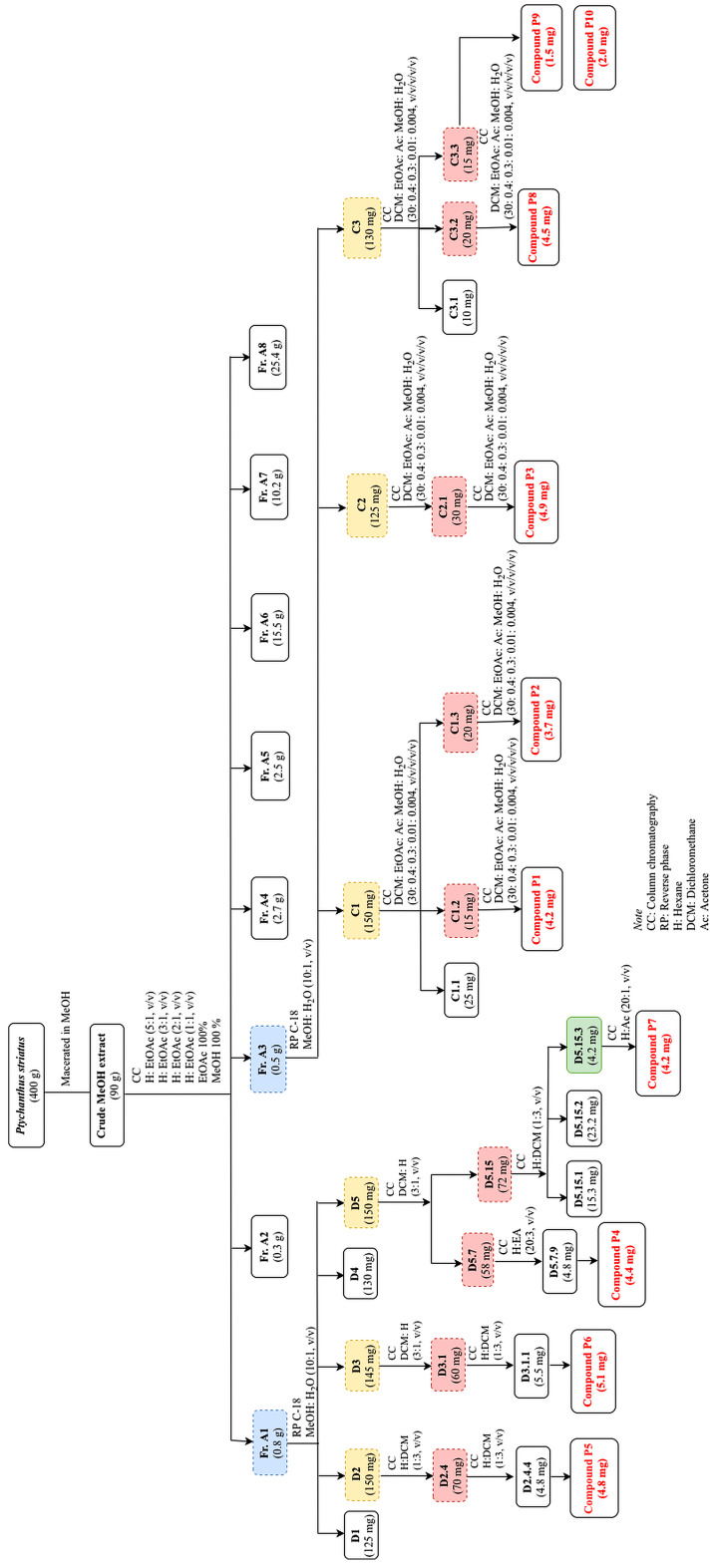
Isolation procedure of compounds **P1**–**P10**.

The dried material of *Ptychanthus striatus* (400 g) was macerated with MeOH at room temperature. After removal of the solvent under reduced pressure, the crude MeOH extract (90 g) was obtained. The MeOH extract was subjected to silica gel column chromatography (CC) and eluted successively with gradients of *n*-hexane–EtOAc (5:1, 3:1, 2:1, and 1:1, *v*/*v*), EtOAc, and MeOH to yield eight fractions, A1–A8. Fraction A1 (0.8 g) was further separated by reversed-phase C18 column chromatography using MeOH–H_2_O (10:1, *v*/*v*) to give five subfractions, D1–D5. Subfraction D2 (150 mg) was chromatographed over silica gel with *n*-hexane–CH_2_Cl_2_ (1:3, *v*/*v*) to afford four fractions, D2.1–D2.4. Fraction D2.4 (70 mg) was further purified by silica gel CC using *n*-hexane–CH_2_Cl_2_ (1:3, *v*/*v*) to obtain subfractions D2.4.1–D2.4.4. Final purification of D2.4.4 (5.8 mg) afforded **P5** (4.8 mg). Subfraction D3 (145 mg) was subjected to silica gel CC eluted with CH_2_Cl_2_–*n*-hexane (3:1, *v*/*v*) to give six fractions, D3.1–D3.6. Further purification of D3.1 (60 mg) over silica gel using *n*-hexane–CH_2_Cl_2_ (1:3, *v*/*v*) afforded D3.1.1 (5.5 mg), which yielded **P6** (5.1 mg). Fraction D5 (150 mg) was separated by silica gel CC using CH_2_Cl_2_–*n*-hexane (3:1, *v*/*v*) to obtain fifteen subfractions, D5.1–D5.15. Subfraction D5.7 (58 mg) was further chromatographed with *n*-hexane–EtOAc (20:3, *v*/*v*) to give nine fractions, D5.7.1–D5.7.9, among which D5.7.9 (4.8 mg) yielded **P4** (4.4 mg). Subfraction D5.15 (72 mg) was purified by silica gel CC using *n*-hexane–CH_2_Cl_2_ (1:3, *v*/*v*) to give three fractions, D5.15.1–D5.15.3. Further purification of D5.15.3 (15.3 mg) with *n*-hexane–acetone (20:1, *v*/*v*) afforded **P7** (4.2 mg). Fraction A3 (0.5 g) was fractionated by reversed-phase C18 column chromatography eluted with MeOH–H_2_O (10:1, *v*/*v*) to give three fractions, C1–C3. Fraction C1 (150 mg) was separated by silica gel CC using CH_2_Cl_2_–EtOAc–acetone–MeOH–H_2_O (30:0.4:0.3:0.01:0.004, *v*/*v*/*v*/*v*/*v*) to afford three fractions, C1.1 (25 mg), C1.2 (15 mg), and C1.3 (20 mg). Purification of C1.2 by silica gel CC with CH_2_Cl_2_–EtOAc–acetone–MeOH–H_2_O (30:0.4:0.3:0.01:0.004, *v*/*v*/*v*/*v*/*v*) yielded **P1** (4.2 mg). Similarly, purification of C1.3 using the same solvent system afforded **P2** (3.7 mg). Subfraction C2 (125 mg) was chromatographed over silica gel with CH_2_Cl_2_–EtOAc–acetone–MeOH–H_2_O (30:0.4:0.3:0.01:0.004, *v*/*v*/*v*/*v*/*v*) to give five fractions, C2.1–C2.5. Further purification of C2.1 (30 mg) using the same solvent system yielded **P3** (4.9 mg). Fraction C3 (130 mg) was separated by silica gel CC using CH_2_Cl_2_–EtOAc–acetone–MeOH–H_2_O (30:0.4:0.3:0.01:0.004, *v*/*v*/*v*/*v*/*v*) to afford three fractions, C3.1 (10 mg), C3.2 (20 mg), and C3.3 (15 mg). Purification of C3.2 with the same solvent system yielded **P8** (4.5 mg). Subfraction C3.3 was further purified by silica gel CC using CH_2_Cl_2_–EtOAc–acetone–MeOH–H_2_O (30:0.4:0.3:0.01:0.004, *v*/*v*/*v*/*v*/*v*) to afford **P9** (1.5 mg) and **P10** (2.0 mg) (Figure 8).

### 4.3. Bioactivity Assay

The inhibitory potential of the compounds against SARS-CoV-2 Mpro was evaluated via a Fluorescence Resonance Energy Transfer (FRET) method. Crude extracts, fractions, and isolated compounds were initially screened at a final sample concentration of 100 µg/mL. The proteolytic enzyme and fluorogenic substrate were prepared separately using the assay buffers. Buffer X consisted of 20 mM Tris-HCl, pH 7.5, 100 mM NaCl, 2 mM DTT, and 0.4 mM EDTA, whereas buffer Y, prepared without DTT, was used for sample preparation. The assay was performed in black 384-well microplates with a final reaction volume of 25 µL. Each reaction mixture contained 10 µL of buffer X, 5 µL of SARS-CoV-2 Mpro solution, and the test sample at the indicated sample concentration. After addition of the test sample and a 10 min pre-incubation period at room temperature, the enzymatic reaction was initiated by adding 5 µL of the fluorogenic FRET substrate solution. Fluorescence kinetics were monitored for 30 min at 60 s intervals using a Tecan Infinite 200 Pro multimode reader (Tecan Group Ltd., Männedorf, Switzerland) with excitation and emission wavelengths of 340 and 430 nm, respectively.

Fluorescence kinetics were tracked for 30 min at 60 s intervals using a Tecan Infinite 200 Pro multimode reader (Ex = 340 nm, Em = 430 nm). The percentage of Mpro inhibition was calculated using the following formula:

$$\mathrm{Inhibition}\;\mathrm{percentage}\;(\%)\;=\;\left(1-\;\frac{S_{s}-\;S_{b}}{S_{c}\;-\;S_{b}}\right)\;\times 100$$

where *S_s_*, *S_c_*, and *S_b_* represent the reaction slopes for the inhibited sample, the controls, and the enzymatic blank, respectively. For potency assessment, IC_50_ values of pure compounds were derived from a 10-point twofold dilution series (0.2 to 80 µM). Data analysis and dose–response curve fitting were performed using GraphPad Prism 8.0.1 via a non-linear regression model with a variable slope. Lopinavir and Ritonavir were used as positive controls [63].

### 4.4. Molecular Docking

The crystal structure of Mpro (PDB ID: 9HAK) was collected from the RCSB Protein Data Bank (PDB). The protein was prepared using the Protein Preparation tool to add and optimize missing hydrogen atoms, while removing unnecessary solvent molecules to ensure the accuracy of interaction predictions [64]. After protein preparation, the native ligand was separated from the protein and redocked using Glide docking at the Standard Precision (SP). The docking protocol was evaluated based on the Root Mean Square Deviation (RMSD), where an RMSD value below 2 Å was considered acceptable [65]. In parallel, the ligands were prepared using LigPrep to add missing hydrogen atoms to the 2D structures (generated using 2D Sketcher) and to optimize their geometries at a pH of approximately 7 ± 2 [64]. After both the protein and ligands were prepared, molecular docking was performed using Glide with SP [66]. Finally, MM-GBSA (Molecular Mechanics–Generalized Born Surface Area) calculations were carried out using the OPLS_4 force field to estimate the binding free energy between the protein and ligands and to further evaluate the reliability of the interactions [4,67]. The binding free energy (${∆G}_{bind}$) was calculated according to the following equation:

$${∆G}_{bind}=∆E_{MM}+∆G_{solv}-T∆S$$

where $∆E_{MM}$ is the molecular mechanics energy, $∆G_{solv}$ is the solvation free energy calculated using the VSGB implicit solvent model, and −T∆S represents the entropic contribution.

## 5. Conclusions

In summary, the present study demonstrated that Vietnamese *Vitex rotundifolia* leaves and the liverwort *Ptychanthus striatus* are valuable sources of structurally diverse secondary metabolites with SARS-CoV-2 Mpro inhibitory potential. Bioassay-guided fractionation of the active extracts led to the isolation of thirteen compounds from *V. rotundifolia* and ten compounds from *P. striatus*, including diterpenoids, triterpenoids, flavonoids, phenolic esters, bibenzyl derivatives, and bisbibenzyl-type metabolites. Most isolated compounds are reported from these species for the first time, and methyl 2-methoxy-6-[2-(4-methoxyphenyl)ethyl]benzoate from *P. striatus* is described as a new natural product. Biological evaluation revealed that the inhibitory activity against SARS-CoV-2 Mpro was not evenly distributed among the isolated compounds. Notably, chrysoplenol D was identified as the most active compound, with an IC_50_ value of 0.08 ± 0.01 µM, indicating markedly stronger inhibition than the positive controls, including ritonavir and lopinavir. These results suggest that the flavonoid framework, particularly with a suitable balance of hydroxy and methoxy substituents, may be favorable for Mpro inhibition. Molecular docking further supported the observed activity by indicating favorable binding of representative active compounds within the Mpro catalytic pocket. Overall, this work provides new phytochemical and biological evidence supporting Vietnamese medicinal plants and bryophytes as promising sources of natural SARS-CoV-2 Mpro inhibitors. Chrysoplenol D and other active flavonoid or bibenzyl-type compounds identified in this study may serve as useful starting points for further mechanistic, structure–activity relationship, and antiviral investigations.

## Figures and Tables

**Table 1:** 2D presentation of interaction of ligands **V9**, **V11**, **V13**, and **P8** with Mpro.

Protein–Ligand Complex	Docking Score (Kcal/mol)	MM-GBSA (Kcal/mol)	Interactions
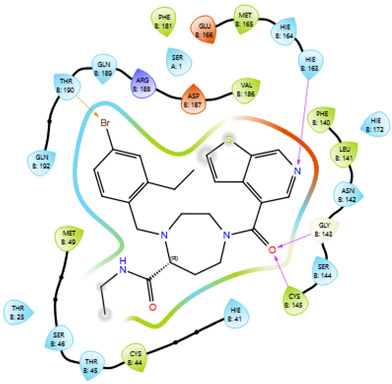 **9HAK–Native ligand**	−9.339	−108.91	H-Bond (HIE41, GLY143, CYS145); Halogen-Bond (GLN192)
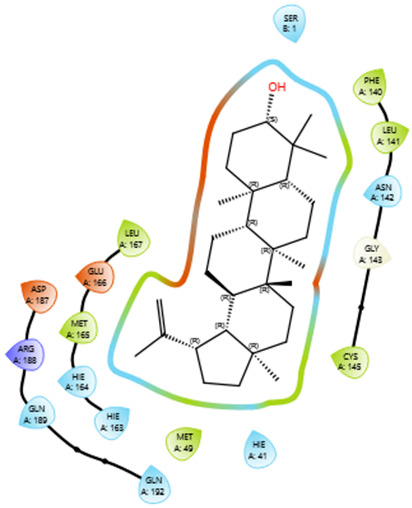 **9HAK–V9**	−4.373	−50.69	-
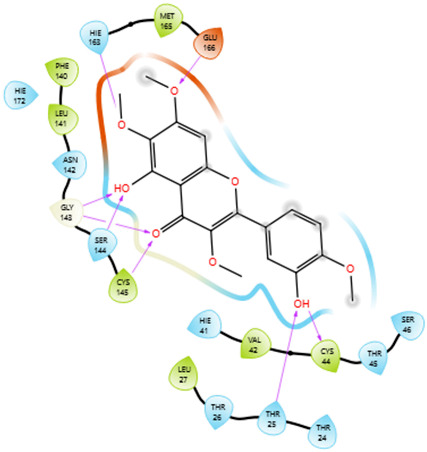 **9HAK–V11**	−6.828	−64.96	H-Bond (THR25, CYS44, GLY143 (2), SER144, CYS145, HIE163, GLU166)
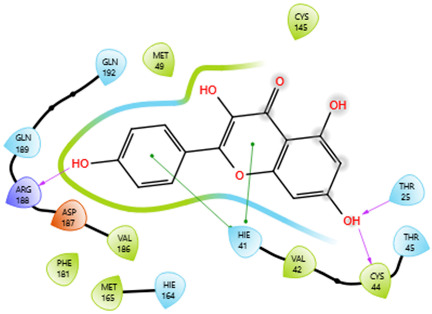 **9HAK–V13**	−7.760	−52.05	H-Bond (THR25, CYS44, ARG188); Pi-pi Stacking (HIE41 (2))
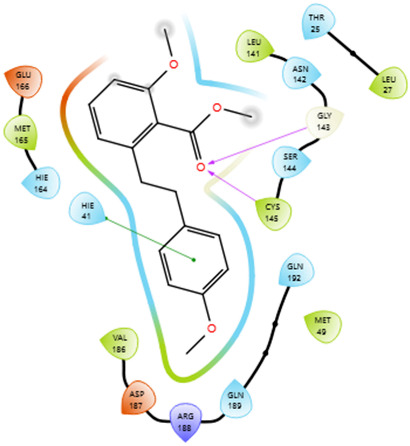 **9HAK–P8**	−6.299	−50.57	H-Bond (GLY143, CYS145) Pi-pi Stacking (HIE41)
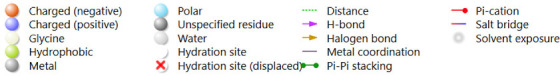			

**Table 2:** Comparison of the NMR spectroscopic data of compound **P5** with those of kigelin.

No	P5		Kigelin [50,51]	
	*δ*_H_ (CDCl_3_, 500 MHz)	*δ*_C_ (CDCl_3_, 125 MHz)	*δ*_H_ (CDCl_3_, 400 MHz)	*δ*_C_ (CDCl_3_ 100 MHz)
1	-	156.0	-	156.1
2	-	135.4	-	135.4
3	-	158.2	-	158.4
4	6.28 (1H, *s*)	102.0	6.28 (1H, *s*)	102.0
5	2.87 (2H, *m*)	34.7	2.86 (2H, *m*)	34.6
6	4.67 (1H, *m*)	75.7	4.66 (1H, *m*)	75.8
7	-	169.7	-	169.8
8	-	102.8	-	102.8
9	-	135.2	-	-
10	1.51 (3H, *d*, *J* = 6.4 Hz)	20.6	1.51 (3H, *d*, *J* = 6.3 Hz)	20.6
1-OH	11.15 (1H, *s*)		11.1 (1H, *s*)	
2-OCH_3_	3.88 (3H, *s*)	60.7	3.87 (3H, *s*)	60.7
3-OCH_3_	3.92 (3H, *s*)	56.1	3.90 (3H, *s*)	56.1

**Table 3:** Comparison of the NMR spectroscopic data of compound **P8** with those of lunularic acid.

No	P8		Lunularic Acid [54]	
	δ_H_ (CDCl_3_, 500 MHz)	δ_C_ (CDCl_3_, 125 MHz)	δ_H_ (CDCl_3_, 500 MHz)	δ_C_ (CDCl_3_, 125 MHz)
1	-	140.2	-	146.5
2	-	-	-	115.4
3	-	156.8	-	163.2
4	6.79 (1H, *d*, *J* = 8.3 Hz)	108.9	6.68 (1H, *d*, *J* = 8.3 Hz)	116.6
5	7.28 (1H, *dd*, *J* = 8.3 Hz)	130.5	7.25 (1H, *dd*, *J* = 8.3 Hz)	134.8
6	6.80 (1H, *d*, *J* = 8.3 Hz)	121.8	6.78 (1H, *d*, *J* = 8.3 Hz)	123.8
7	-	169.7	-	174.8
α	2.82–2.83 (2H, *m*)	36.2	3.14 (2H, *t*, *J* = 8.5 Hz)	39.2
β	2.82–2.83 (2H, *m*)	36.9	2.76 (2H, *t*, *J* = 8.5 Hz)	40.9
1′	-	131.9	-	135.0
2′, 6′	7.10 (1H, *d*, *J* = 8.5 Hz)	129.4	7.00 (1H, *d*, *J* = 8.6 Hz)	130.8
3′, 5′	6.84 (1H, *d*, *J* = 8.5 Hz)	113.9	6.70 (1H, *d*, *J* = 8.6 Hz)	116.5
4′	-	157.9	-	156.8
7-OCH_3_	3.94 (3H, *s*)	52.4	-	-
3-OCH_3_	3.85 (3H, *s*)	56.1	-	-
4′-OCH_3_	3.80 (3H, *s*)	55.4	-	-

## Data Availability

Data are available upon requests.

## Supplementary Materials

The following supporting information can be downloaded at: https://www.mdpi.com/article/10.3390/molecules31122009/s1, Figure S1. ^1^H NMR spectrum of compound P8; Figure S2. ^13^C NMR spectrum of compound P8; Figure S3. HMBC spectrum of compound P8; Figure S4. HMQC spectrum of compound P8; Figure S5. NOESY spectrum of compound P8; Figure S6. ESI mass spectrum of compound P8.
