# Supplementary material for: Bioassay-Guided Phytochemical Investigation of Vietnamese Vitex rotundifolia Leaves and the Liverwort Ptychanthus striatus as Sources of SARS-CoV-2 Main Protease Inhibitors
Source: Molecules. 2026 Jun 8;31(12):2009. doi: 10.3390/molecules31122009 (PMC13305993; doi:10.3390/molecules31122009)
Supplement: Supplementary file 1 [file molecules-31-02009-s001.zip › molecules-4336482-supplementary.pdf]

Supporting information

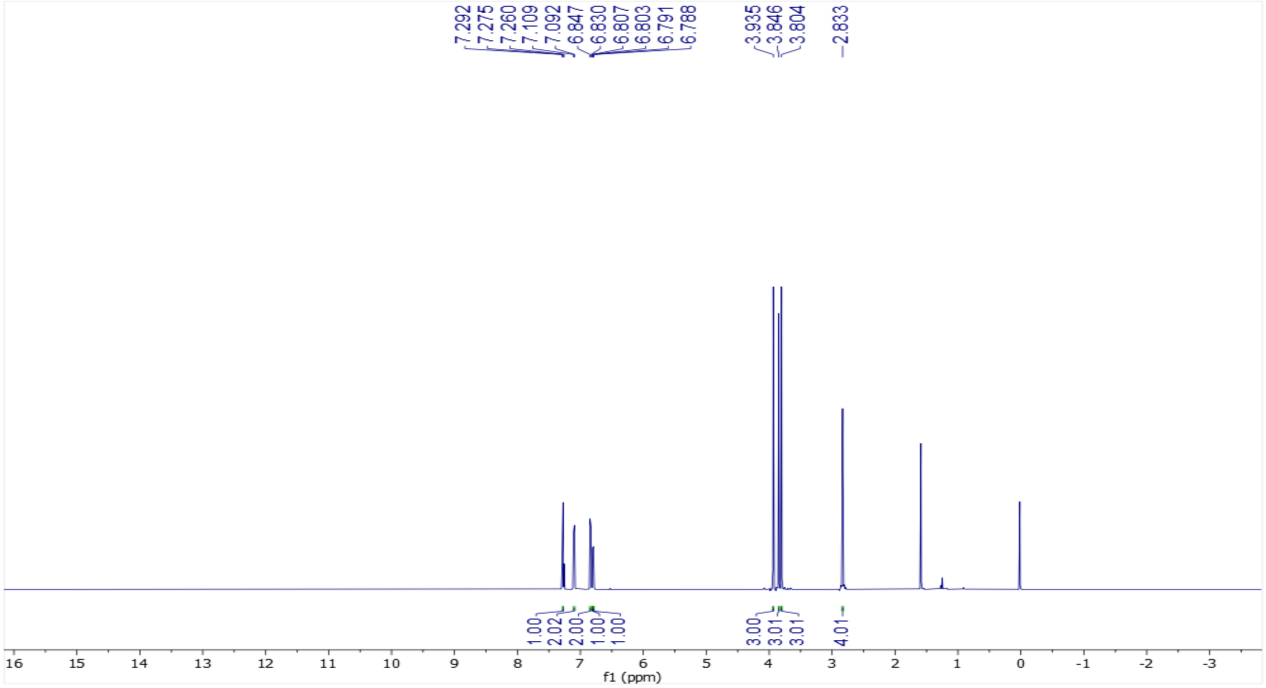

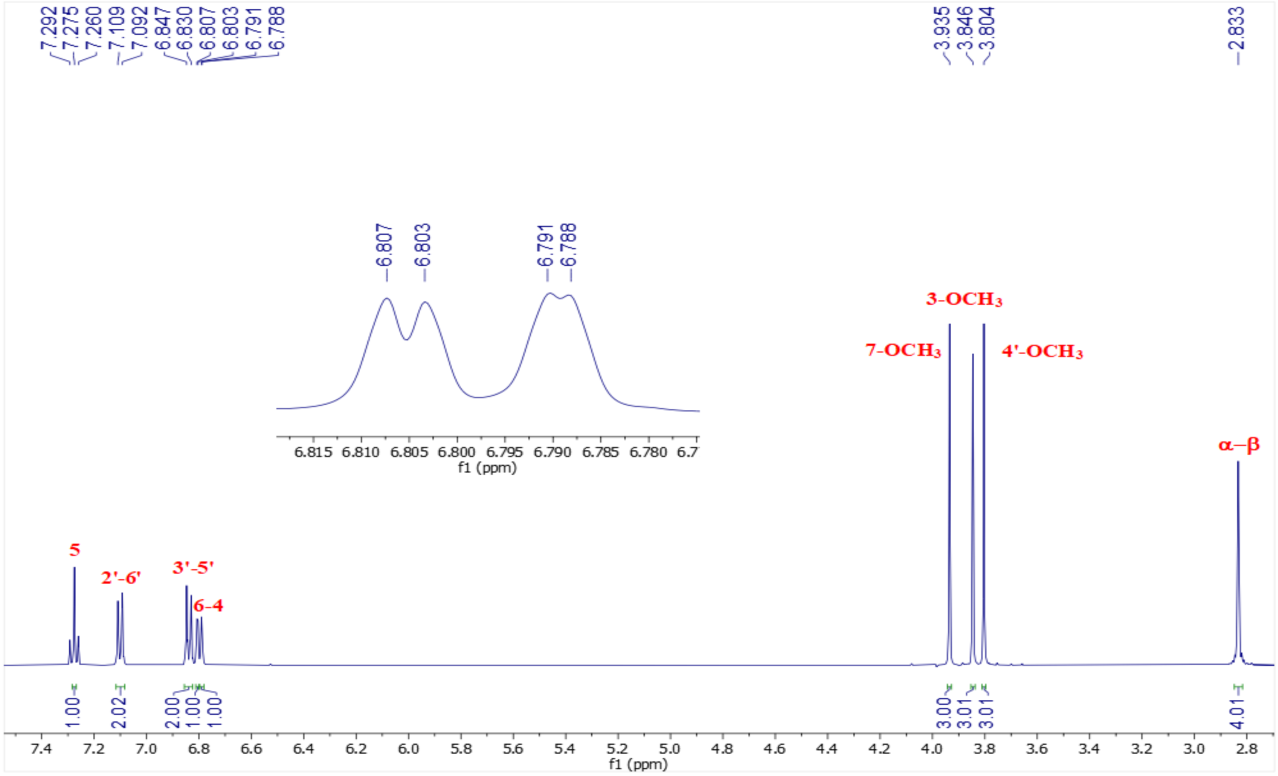

Appendix 1. ^{1}H NMR spectrum of compound P8

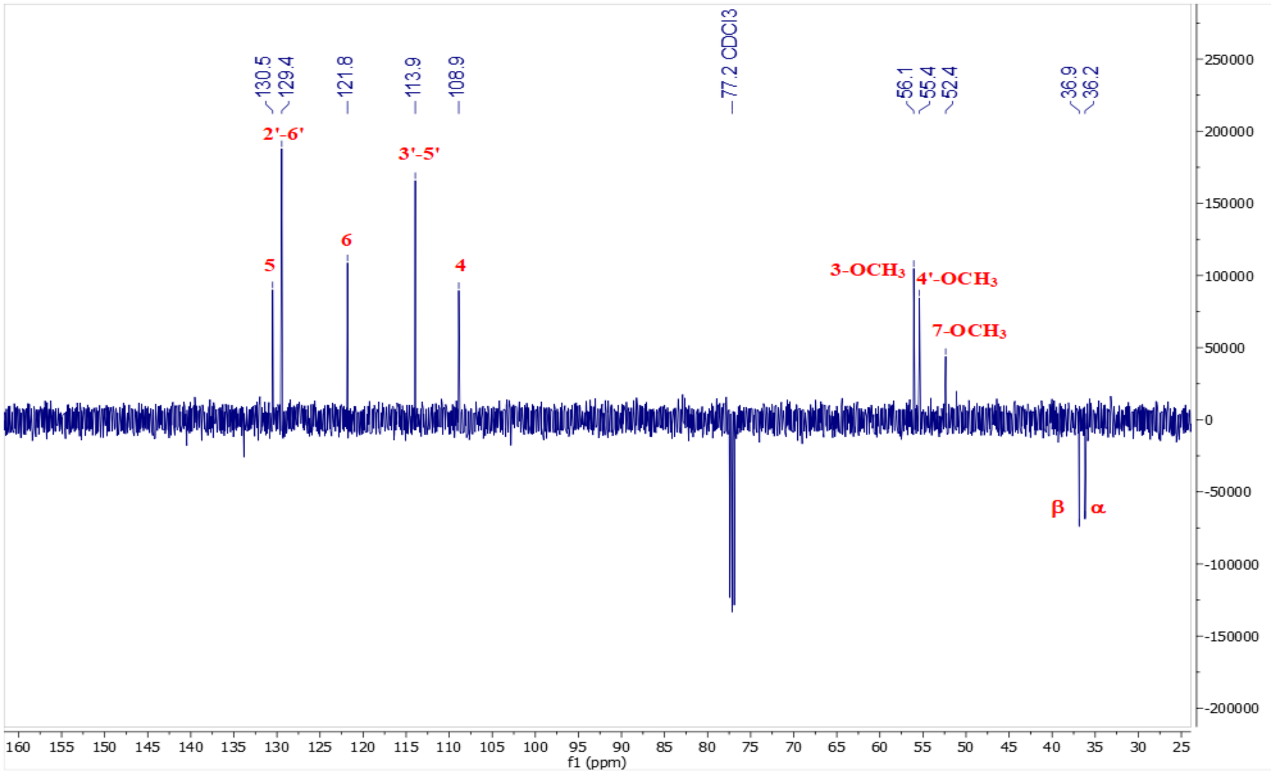

Appendix 2. ^{13}C NMR spectrum of compound P8

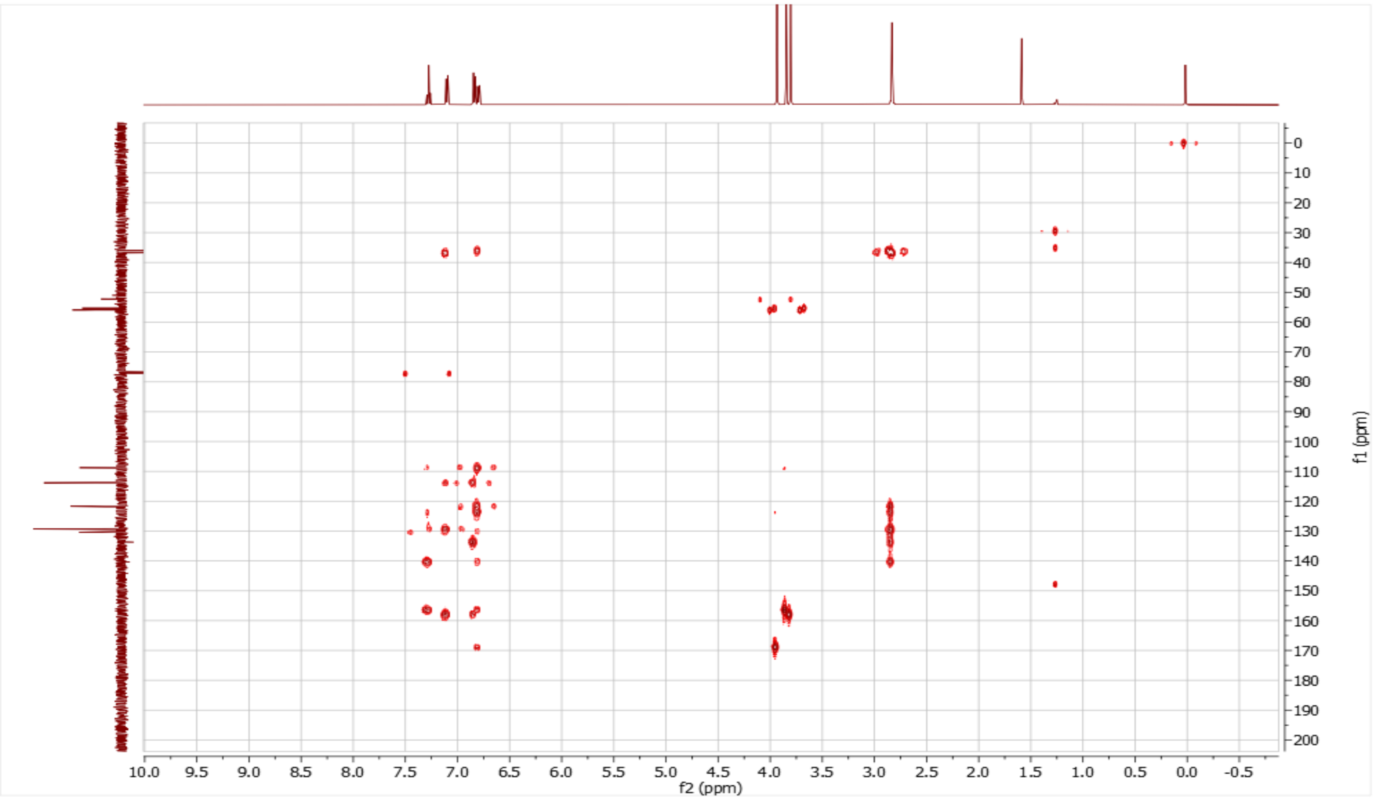

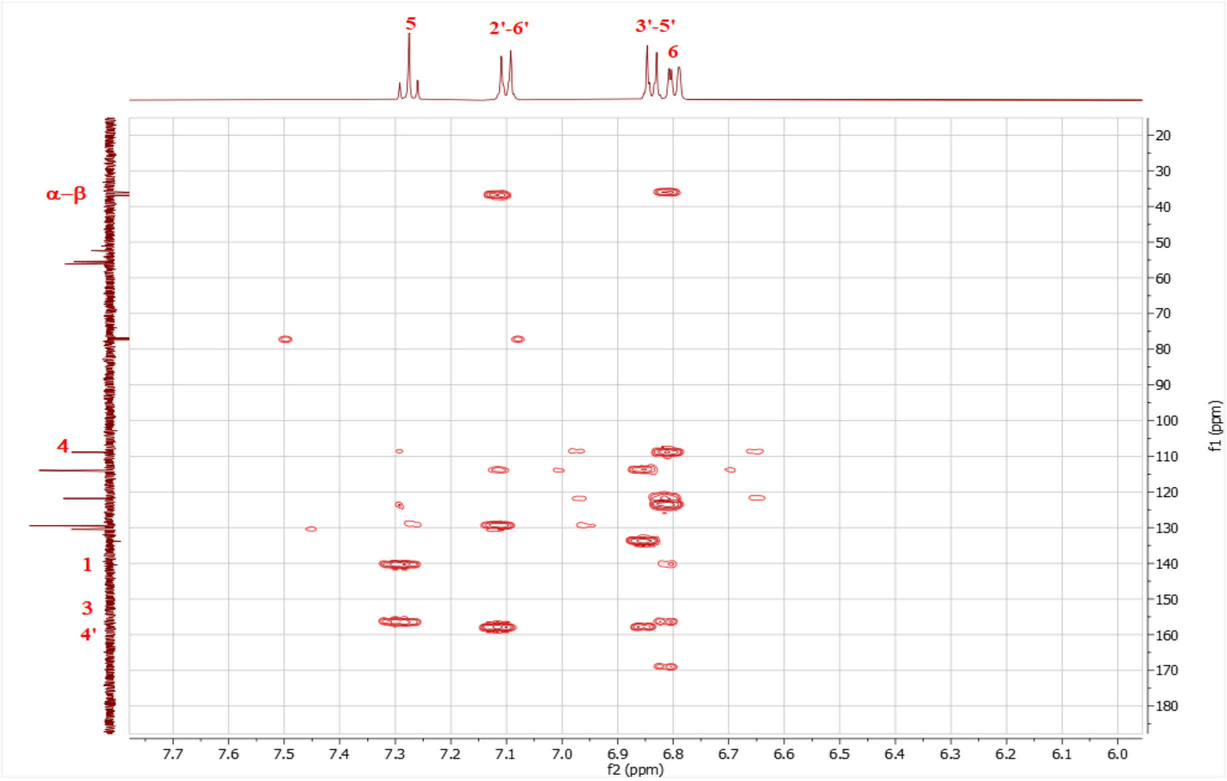

Appendix 3. HMBC spectrum of compound P8

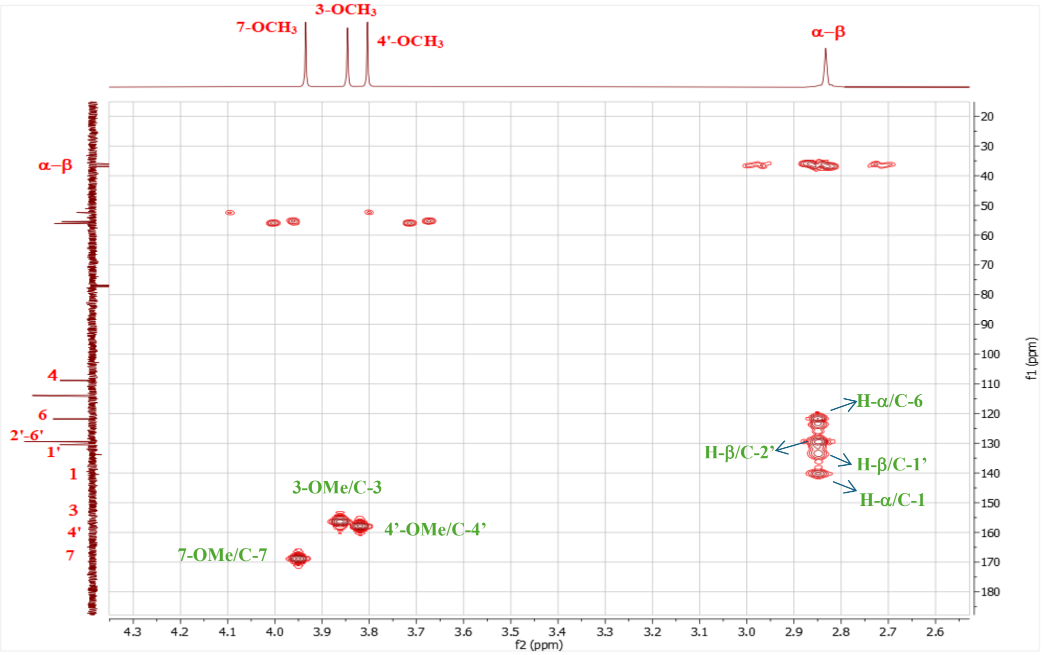

Appendix 3. HMBC spectrum of compound P8 (expanded)

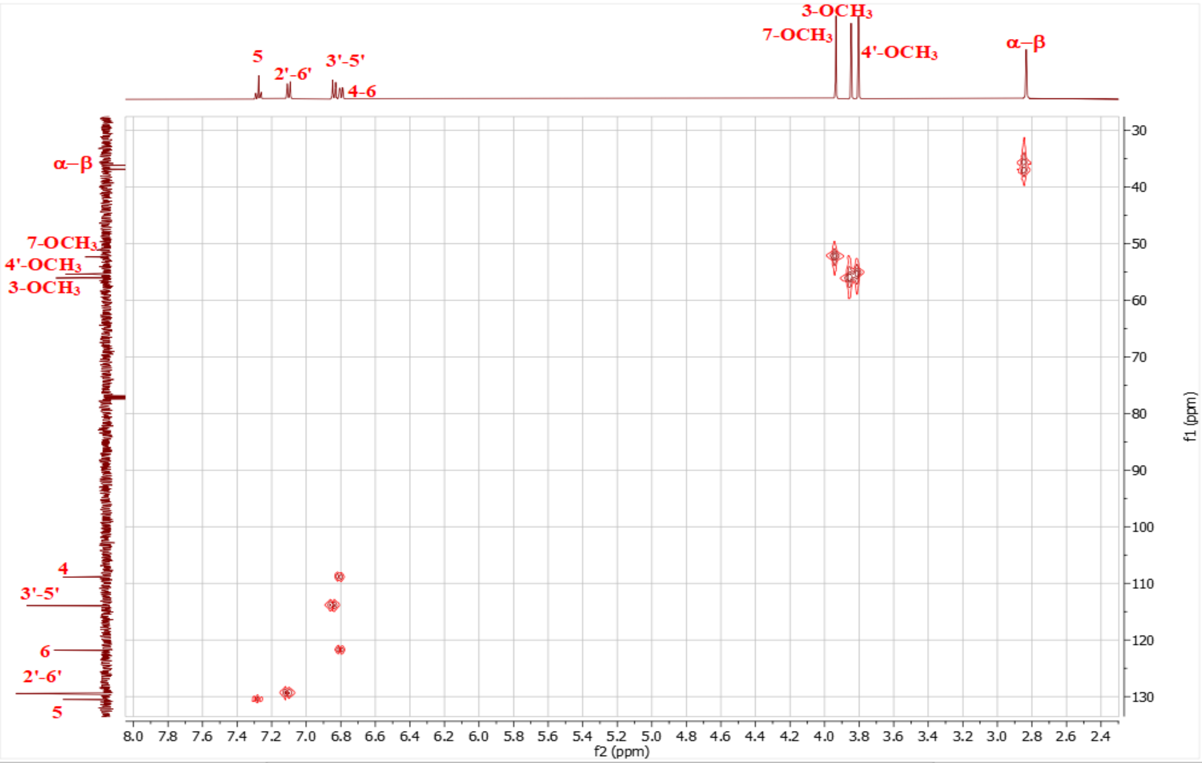

Appendix 4. HMQC spectrum of compound P8

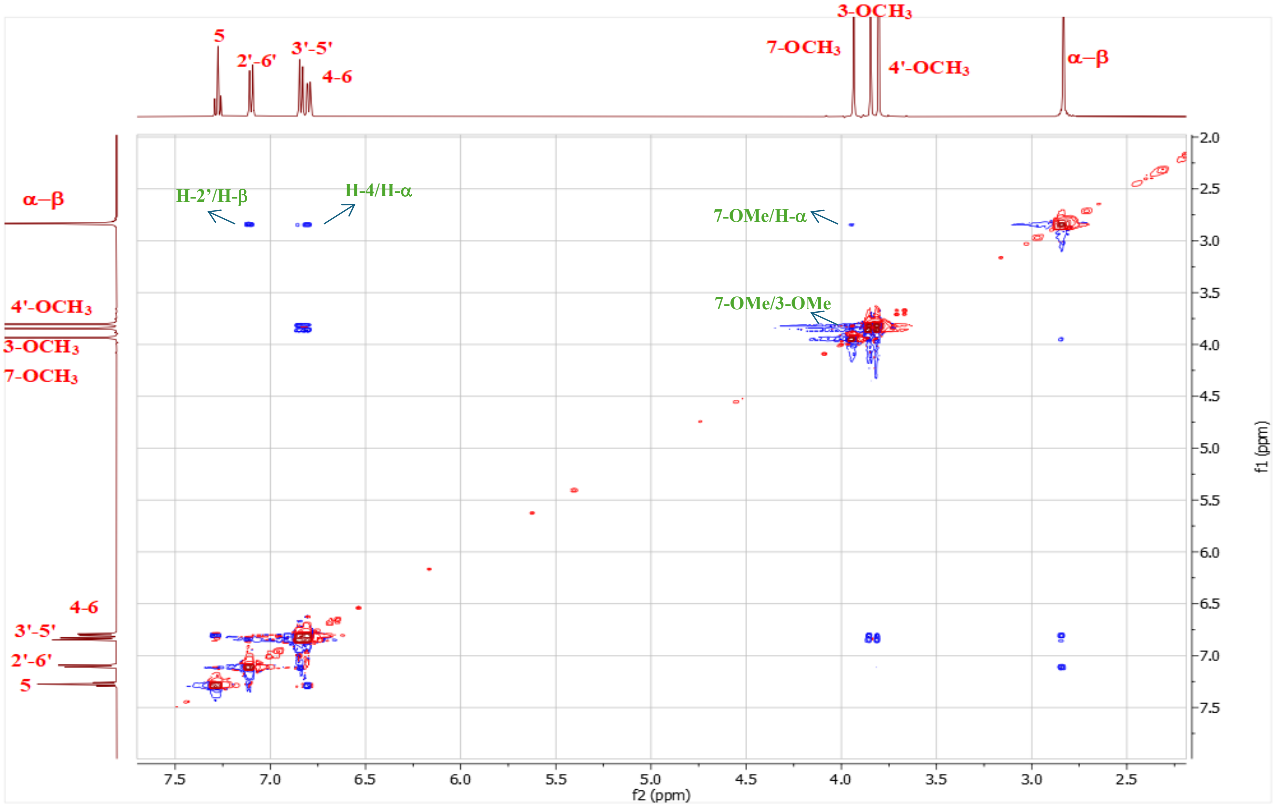

Appendix 5. NOESY spectrum of compound P8

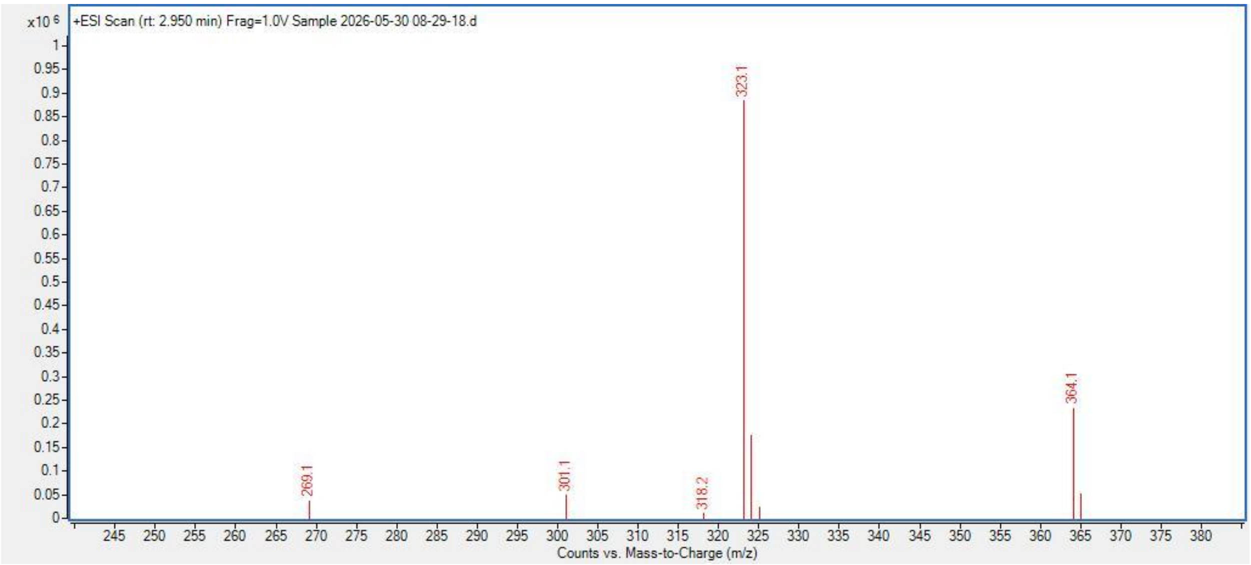

Appendix 6. ESI mass spectrum of compound P8
